# DIZZiness treatment through implementation and clinical strategy Tactics-2 (DIZZTINCT-2) project—a clinical trial protocol

**DOI:** 10.1186/s13063-025-09055-7

**Published:** 2025-09-29

**Authors:** William J. Meurer, Stacy Park, Huong Nguyen, Silvia R. Paz, Molly O. Jancis, Julliane Bacerdo, Aileen S. Baecker, Prasanth Manthena, Navdeep S. Sangha, Chengyi Zheng, Lawrence C. An, Terry D. Fife, Adam L. Sharp, James F. Burke, Kevin A. Kerber

**Affiliations:** 1 Departments of Emergency Medicine and Neurology, University of Michigan, Ann Arbor, MI USA; 2 Department of Research & Evaluation, Kaiser Permanente Southern California, Pasadena, CA USA; 3 Department of Research & Evaluation, Kaiser Permanente, Pasadena, CA USA; 4 Kaiser Permanente, Panorama City, CA USA; 5 Kaiser Permanente, Pasadena, CA USA; 6 SCPMG, Kaiser LAMC Neurology, Los Angeles, CA USA; 7 Southern California Permanente Group and Kaiser Permanente Bernard J Tyson School of Medicine, Los Angeles, CA USA; 8 Division General Medicine, Department Internal Medicine, University of Michigan, Ann Arbor, MI USA; 9 Barrow Neurological Institute and University of Arizona College of Medicine, Phoenix, AZ USA; 10 Galvan Health, Jackson Hole, WY USA; 11 Department of Neurology, Ohio State University, Columbus, OH USA

**Keywords:** Vertigo, Emergency department, Stepped-wedge, Randomized controlled trial

## Abstract

**Background:**

The evaluation and management of acute vertigo presentations is challenging for both patients and physicians. Benign paroxysmal positional vertigo (BPPV), acute unilateral vestibulopathy (e.g., vestibular neuritis), and stroke are priority diagnostic considerations in this circumstance. Existing evidence can be used to guide the diagnosis and treatment, however high value care opportunities—such as the Dix-Hallpike test (DHT), canalith repositioning maneuver (CRM), and gaze stabilization exercises (GSE)—are often underused, while neuroimaging studies are often overused.

**Methods:**

This trial contains a health system focused stepped wedge intervention and an embedded individually patient randomized clinical trial. The study will start with a 6-month pre-intervention period. This will be followed by staggered intervention at the engaged EDs in 11 waves and then an approximately 6-month post-intervention period. Concurrently, patients will be recruited before and after the physician level intervention is implemented at each ED. Enrolled participants will complete baseline survey and then be randomized individually, stratified by sex, age, and medical center, to the intervention or control arm patient materials using central computerized randomization. The intervention arm will be sent intervention materials and the control arm will be sent the hospital’s standard post-discharge materials. The primary outcome of the physician-based part of the trial is use of evidence-based care practices during the index ED visit. The primary outcome of the patient focused part of the trial is the dizziness handicap index over 4 weeks.

**Discussion:**

The DIZZTINCT-2 trial addresses key areas of uncertainty in how to improve the care of emergency department patients with acute vertigo. In addition, follow up data on how much and how fast patients improved was needed. DIZZTINCT-2 will address these key knowledge gaps efficiently.

**Trial registration:**

Clinicaltrials.gov NCT05634902. Registered on November 2022.

## Administrative information

**Table:** 

Title {1}	DIZZiness Treatment through ImplementatioN & Clinical strategy Tactics-2 (DIZZTINCT-2) Project–A clinical trial protocol
Trial registration {2a and 2b}.	clinicaltrials.gov identifier NCT05634902
Protocol version {3}	Version 2–Approved April 2024
Funding {4}	National Institutes of Health: R01DC012760
Author details {5a}	See above
Name and contact information for the trial sponsor {5b}	Kevin A. Kerber, Department of Emergency Medicine, The Ohio State University, Columbus OH Kevin.Kerber@osumc.edu
Role of sponsor {5c}	The funding body has no role in collection, analysis, and interpretation of data and did not have a direct role in the writing of the manuscript.

## Introduction

### Background and rationale {6a}

#### Brief summary

DIZZTINCT-2 focuses on benign paroxysmal positional vertigo (BPPV), acute unilateral vestibulopathy (e.g., vestibular neuritis), and stroke, which have established evidence-based practices for evaluation and management: the Dix-Hallpike test (DHT), Canalith repositioning maneuver (CRM), and gaze stabilization exercise (GSE), and a stroke risk assessment. The premise is that these practices have not been adequately disseminated to ED providers and patients, leading to missed opportunities for effective and efficient care delivery. Emergency department providers have ranked dizziness as a top priority for decision support [10].

Building on the success of the intervention used in DIZZTINCT-1, which was associated with an increased safe use of BPPV tests and treatments and reduced use of head CTs in dizziness visits, DIZZTINCT-2 aims to scale-up the reach of the materials to non-academic site physicians, enhance the clinical topics, and add patient-self-management resources. DIZZTINCT-2 also evaluates patient-centered outcomes and includes both English and Spanish versions of patient materials [29].

#### Rationale

BPPV is the most common peripheral vestibular disorder, with a lifetime prevalence of ~10% [54]. BPPV accounts for 8% of individuals with moderate or severe dizziness [54]. Despite being labeled “benign,” BPPV patients experience significant inconveniences and disabilities during symptomatic periods [35, 54]. Nearly 1 in 4 BPPV patients stop driving, 1 in 3 miss work, and more than 3 in 4 seek medical consultation [54].

The DHT and CRM are the gold standard test and treatment for BPPV, supported by numerous randomized controlled trials and systematic reviews [4, 5, 13, 20, 21, 53, 56]. Despite this, DHT and CRM are underused, with 94–96% of the target population not receiving these procedures, due to misconceptions and difficulty remembering how to perform them [27, 30, 38, 39, 43, 54]. The DHT and CRM are for posterior canal BPPV, the most common type. Other tests and treatments are available for horizontal BPPV.

Optimal use of DHT and CRM can reduce unnecessary head CTs, which are often performed in 30–40% of BPPV visits, exposing patients to radiation, higher costs, and longer stays [27, 29, 33, 39]. Correct use of DHT also aids in diagnosing other disorders needing different treatments, such as vestibular neuritis and stroke [31].

#### Identification of BPPV: the Dix-Hallpike test (DHT)

**Fig. 1:**
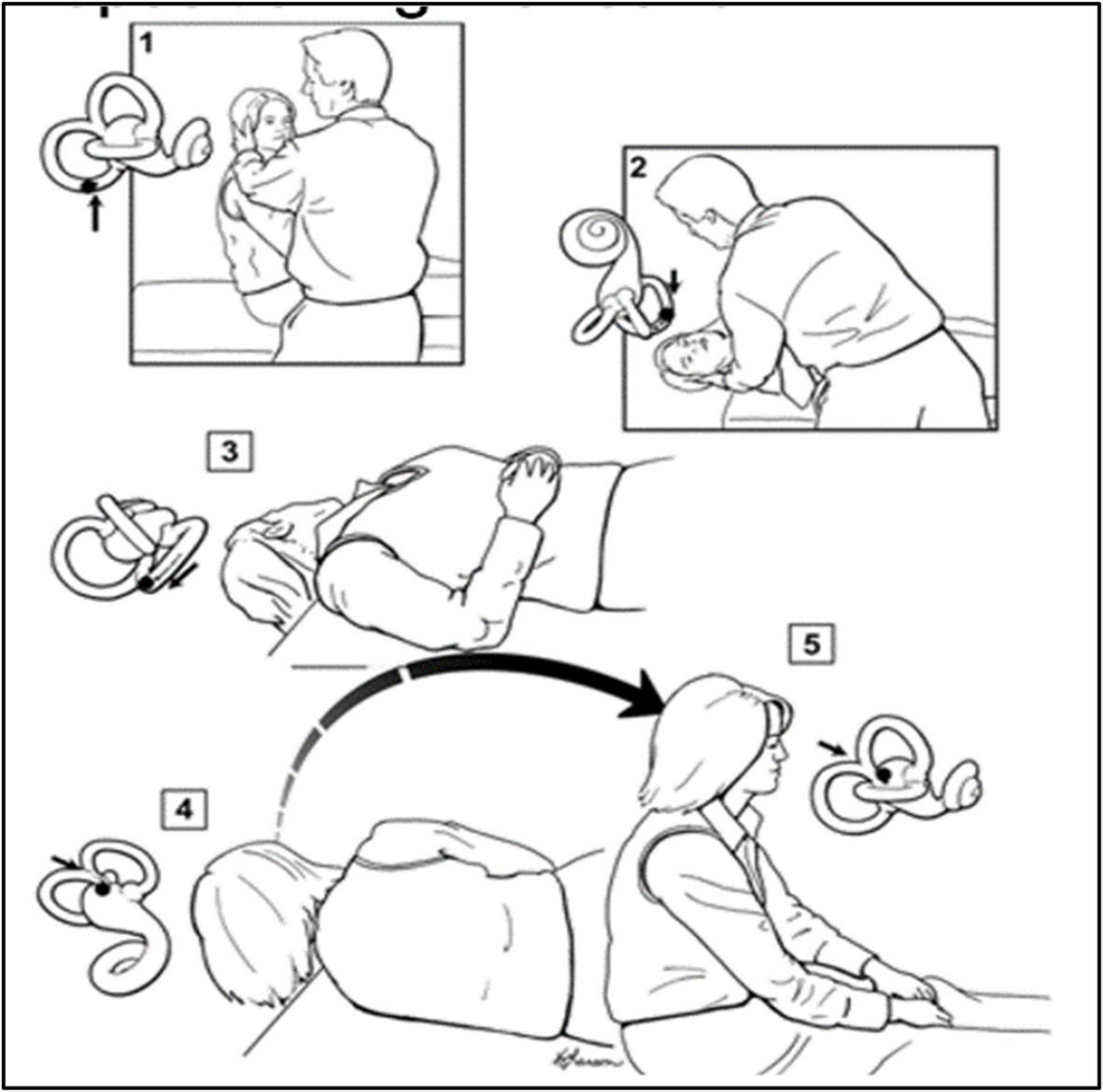
The Dix-Hallpike test

The DHT is the gold standard test for BPPV [4, 13]. It is a simple bedside test (Fig. 1). A positive test is indicated by up-beating and torsional nystagmus lasting about 10–20 s. However, physicians may misinterpret results, calling the test positive for symptoms rather than nystagmus, or misdiagnosing BPPV with any observed nystagmus pattern [42, 45, 50]. Nystagmus can also be observed with other disorders on positional testing: vestibular neuritis has horizontal and persistent nystagmus typically also observed prior to positional testing,central disorders can have persistent down-beating nystagmus.

#### Treatment of BPPV: the canalith repositioning maneuver (CRM)

The CRM is the evidence-based, multidisciplinary guideline-concordant treatment for BPPV [4]. The CRM is designed to move canaliths around and out of the posterior canal into to the central chamber of the inner ear, thereby resolving positional vertigo. The first two steps of the CRM are the same as the DHT. If the DHT is positive, three additional steps move the particles, via the influence of gravity, out of the canal [13].

#### Relevance and priority for this study

The topic is highly impactful due to the number of affected patients (BPPV lifetime prevalence is ~10% [54]), the efficacy of CRM [4, 13], and the potential for improvement in healthcare efficiency [32, 33, 41, 45, 54]. The study brings together experts from multiple disciplines to address a top priority for decision support associated with high unnecessary testing and low use of evidence-based practices [10, 32, 33, 41, 44, 54]. The project aims to improve the effectiveness and efficiency of care for BPPV and other vestibular disorders.

Frontline physicians have expressed a strong need for vertigo support, ranking it as the #1 topic in adult ED presentations [10]. BPPV is common, identifiable, and treatable at the bedside without laboratory or imaging studies, which are discouraged in guideline statements [4]. ED physicians have advocated for BPPV processes, stopping an ED-based trial for ethical reasons due to the effect size at interim analysis [8]. Our preliminary studies indicate high demand for BPPV intervention.

#### Supporting data

The scientific premise of DIZZTINCT-2 is based on prior work in DIZZTINCT-1 which defined the problems, created an implementation strategy and materials, and demonstrated that providers adopted the strategy [29]. DIZZTINCT-1 aimed to increase the use of DHT and CRM and promote a BPPV-centric approach to dizziness. The strategy was guided by the Consolidated Framework for Implementation Research (CFIR) and informed by ED provider interviews to identify barriers and facilitators to DHT and CRM use [9, 30].

The DIZZTINCT-1 stepped-wedge RCT found that the implementation strategy increased DHT or CRM use (odds ratio 2.44; 95% CI 1.42 to 4.20; *n* = 7635 visits) [29]. The DHT or CRM use increased from 7% in pre-intervention visits to 17% in post-intervention visits in the estimated target population with a high probability of BPPV diagnosis (1.5 to 3.5% among all dizziness visits) [29]. The strategy was safe, reducing head CT use (from 44 to 37%) and showing a trend toward lower hospital admission. No serious adverse events were related to DHT or CRM, and non-serious adverse events (nausea, vomiting, headache) were uncommon (<5% of visits) and resolved or improved. There was also a trend toward a lower rate of subsequent strokes after ED visits in intervention visits compared with control visits [29]. Qualitative analysis of provider interviews revealed that providers often had positive experiences using the materials and performing the DHT and CRM [29].

#### Limitations of DIZZTINCT-1 for scaling up

DIZZTINCT-1 had limitations that need to be addressed before widespread dissemination [29]. Low provider participation at non-academic facilities was a major issue. Academic facilities had higher participation presumably due to the learning culture and routine education activities. Providers suggested refining and enhancing the strategy to make it more convenient and resourceful. DIZZTINCT-1 did not assess patient outcomes, which will be evaluated in DIZZTINCT-2. DIZZTINCT-2 enhances the CME session, expands clinical topics, incorporates patient-oriented materials, and captures patient-oriented outcomes during follow-up.

#### Acute unilateral vestibulopathy (vestibular neuritis) and stroke-dizziness

DIZZTINCT-2 includes detailed information on vestibular neuritis and stroke-dizziness. It incorporates evidence-based methods to distinguish vestibular neuritis from stroke, including the BE-FAST guide to identify stroke warning signs (balance issues, eye problems, facial drooping, arm weakness, speech difficulty, and acute onset) and a nystagmus assessment with video examples for central patterns of nystagmus and vascular risk assessment [1, 3, 7, 11, 31, 55, 57]. The algorithm also guides physicians on using the evidence-based treatments for vestibular neuritis: gaze-stabilization and balance/walking exercises [19].

#### Patient materials for management of dizziness

Patient materials may be important to improve the delivery of evidence-based dizziness management and patient outcomes. In DIZZTINCT-1, providers suggested that patient materials could improve the value and use of resources. BPPV self-management is effective. However, previous evaluations of available online resources for BPPV showed that while they generally describe steps accurately, patients often find instructions confusing [4, 26]. DIZZTINCT-2 includes patient-oriented BPPV self-management educational resources, evaluated positively by patients on measures of attitudes, self-efficacy, and social norms [28]. Self-management of unilateral vestibulopathy (vestibular neuritis) with gaze-stabilization and balance/walking exercises is also supported by a multidisciplinary guideline [19].

#### Summary

DIZZTINCT-2 builds on the findings of DIZZTINCT-1 and seeks to broaden its reach by addressing opportunities to optimize care efficiencies and patient outcomes for dizziness in the ED. This project has the potential to lead to lasting positive changes in the clinical care of patients with dizziness. By expanding the scope and evaluating patient outcomes, DIZZTINCT-2 aims to effectively implement evidence-based management practices for vestibular disorders.

### Objectives {7}

To improve the current management of dizziness in the ED and patient outcomes by using a provider- and patient-based educational intervention and implementation strategy focused on vestibular disorders.

#### Objective 1 (ED-level)

To determine the impact of an enhanced BPPV-centric implementation strategy on DHT/CRM performance in dizziness visits, at academic and nonacademic EDs, using a randomized stepped-wedge design. The strategy targets physicians and includes enhanced provider materials, patient-oriented resources, local champions, an online educational session, and reminder system.

#### Objective 2 (patient-level)

To evaluate clinical outcomes associated with the implementation strategy using both a stepped-wedge ED-level strategy and an embedded randomized clinical trial of a patient-level dissemination strategy. The patient-level strategy includes materials delivered to SPIRIT guidance: Specific objectives or hypotheses.

### Trial design {8}

We will conduct a stepped-wedge, randomized clinical trial of a multi-faceted educational and care-process based intervention designed to improve the guideline-concordant care of patients with BPPV in the emergency department. The overall design includes an ED-level implementation and embedded, patient-level dissemination strategies which will allow evaluation of both implementation and effectiveness outcomes. Our study design and proposed methods are aligned with methodological standards in a stepped wedge, randomized clinical trial. The overall study period will be approximately 24 months. The study will start with a 6-month pre-intervention period. This will be followed by staggered intervention at the 15 EDs in 11 waves (some medical centers will be paired based on the medical service area) and then an approximately 6-month post-intervention period. The order that each ED receives the intervention will be randomized. The intervention will be provided as a complete package during the month each hospital is randomized. Concurrently, patients will be recruited before and after the physician level intervention is implemented at each ED. Enrolled participants will complete a survey to obtain baseline information and then randomized individually, stratified by sex, age, and medical center, to the intervention or control arm using central computerized randomization. The intervention arm will be sent intervention materials via patient specified preferred electronic communication. The control arm will be provided with the hospital’s standard post-discharge materials.

## Methods: participants, interventions and outcomes

### Study setting {9}

We will examine the interventions during the course of providing usual care for dizziness visits at Kaiser Permanente Southern California Emergency Departments (EDs). KPSC is a large integrated health care system that provides comprehensive health care services for approximately 1 in 5 Southern California residents (~4.8 million members). Kaiser Permanente is the largest real-world care setting in the nation and an ideal environment for population based clinical and health services research. The member population is socioeconomically diverse and broadly representative of the racial/ethnic groups living in Southern California. The organization’s model of care and information infrastructure allows the generation of evidence to support practice decisions and improve clinical outcomes. The electronic medical record (EMR) system, implemented at all 15 KPSC medical centers by 2006, contains complete visit-level data on health services, medications, and outcomes. We have confirmed EMR availability of ED visit data since 2007 [15]. The billing system can identity health service use by members at non-KPSC facilities. Vital statistics are linked to all members and, death can be ascertained regardless of membership status after the index visit.

### Eligibility criteria {10}

#### ED-level

- Age 18 years or older
- Dizziness as primary reason for visit and/or discharge diagnosis
- KPSC membership in last 31 days
- No level 1 trauma code
- Not a prisoner

#### Patient-level (additional to ED-level above)

- Discharged home from ED or observation
- Dizziness as primary discharge diagnosis (dizziness, vertigo, lightheadedness, or imbalance)
- English or Spanish speaker
- Not cognitively impaired
- Not previously enrolled
- Capacity to consent—assessed by the Older Adults’ Capacity to Consent to Research (OACCR) scale

### Who will take informed consent? {26a}

We will obtain a waiver of consent to screen medical records and to abstract information from visits meeting the inclusion/exclusion criteria. The risk from medical record review is no more than minimal risk.

Participants in the patient level intervention activities will undergo formal informed consent and electronically sign an informed consent form. Consent will be obtained using standard language and content as approved by the IRB and adjusted to the specific circumstances of this project. Consent will be available using a self-service survey sent to participants via email or text. The self-directed process has opportunity to call study team for questions.

### Additional* consent provisions for collection and use of participant data and biological specimens {26b}*

We do not collect biological specimens and data use is considered in the main consent.

## Interventions

### Explanation* for the choice of comparators {6b}*

#### ED level

The intervention is described further in 11a below. The standard care case is existing usual ED processes.

#### Patient level

The intervention is described further in 11a below. Patients allocated to control (standard care) receive routine ED discharge instructions and referral to KPSC general resources in dizziness.

### Intervention description {11a}

#### ED level

The overall education intervention is delivered at the medical center level. These consist of educational materials, educational sessions, checklists, preparing local champions, available dot phrases, and designing reminder systems. Educational materials will consist of a mobile friendly website, and printed posters and notecards. A local champion will be recruited and prepared to help with notification of providers, dissemination of print materials (posters, cards), notification of decision aids, and answering questions. Although investigators will use a standard approach and some components will be fixed local champions can adapt distribution and notification of materials.

#### Patient level

The intervention components are the patient-oriented website about the test and treatment for BPPV and gaze stabilization exercises for vestibular neuritis. The videos will include background information on BPPV and vestibular neuritis and video examples of self-performance of the Dix-Hallpike test, canalith repositioning maneuver, gaze stabilization exercises, and walking and balance exercises. Prior research has established the safety and potential effectiveness of self-treatment using the CRM and gaze-stabilization exercises.

SPIRIT guidance: Interventions for each group with sufficient detail to allow replication, including how and when they will be administered.

### Criteria for discontinuing or modifying allocated interventions {11b}

We did not set any formal criteria for discontinuing either the provider or patient facing interventions because they were low risk.

### Strategies to improve adherence to interventions {11c}

We plan to assess implementation fidelity in order to monitor adherence during the trial and in plans to give recommendations for implementation in other settings. Our fidelity assessments have both quantitative and qualitative components to capture clinical and strategy fidelity. The quantitative data is the use of the educational session, print materials, website, app, checklist, and electronic medical record reminders. The use data will directly inform the interpretation of necessary components. Qualitative data will be obtained from semi-structured interviews with key stakeholders. We will sample individuals to capture a range of experiences with the materials and strategy (e.g., high frequency resources users, low frequency resource users, local clinical leaders). We will use an interview guide developed with our multidisciplinary team to assess training uptake, adherence to content, quality of use in practice, barriers to use in practice, resource dosage, satisfaction, and site adaptations. Interviews with local champions will specifically seek to capture variability in the approaches to provider notifications. Regarding, participant (patient-level) follow up—we use a variety of communication methods (email, phone, text) and financial incentives to promote adherence to the data collection.

SPIRIT guidance: Strategies to improve adherence to intervention protocols, and any procedures for monitoring adherence (e.g., drug tablet return, laboratory tests).

### Relevant concomitant care permitted or prohibited during the trial {11d}

The hospital staff and enrolled patients are permitted to engage in any and all routine necessary concomitant medical treatments. We do not define any prohibitions.

### Provisions for post-trial care {30}

The trial does not have any formal procedures for post-trial care.

### Outcomes {12}

#### Primary outcome

##### Physician level

The primary outcome measure for the ED-level implementation strategy is DHT or CRM performance documentation (timing: during initial ED visit only).

##### Patient level

The primary outcome measure for the patient-level intervention is the dizziness handicap inventory (DHI) patient reported outcome (PRO) and will be obtained at weekly intervals for 1 month.

#### Secondary, exploratory, safety outcomes, and adverse events

##### Physician level

Secondary healthcare utilization related outcomes include the following:

- Brain imaging (head CT, brain MRI)
- Hospital admission
- Length of stay in the ED

Safety outcomes include the following:

- Stroke hospitalizations within 30 days of ED/hospitalization
- Return visits to ED/urgent care/hospitalization for dizziness within 30 days

Adverse events related to the use of DHT/CRM (see Section 11)

##### Patient level

Secondary and exploratory outcomes include the following:

- Days-Out-Of-Role
- Patient Global Impression of Change
- World Health Organization Well Being Index
- Patient Health Questionnaire (PHQ)−4
- DHT/CRM use
- Gaze-stabilization use
- Satisfaction with the materials

##### Safety outcomes

- Stroke hospitalizations within 30 days of ED/hospitalization
- Return visits to the ED/urgent care/unscheduled hospitalization for dizziness within 30 days

Adverse events within 30 days related to the use of DHT/CRM, gaze stabilization exercises, walking exercises

### Participant timeline {13}

**Fig. 2:**
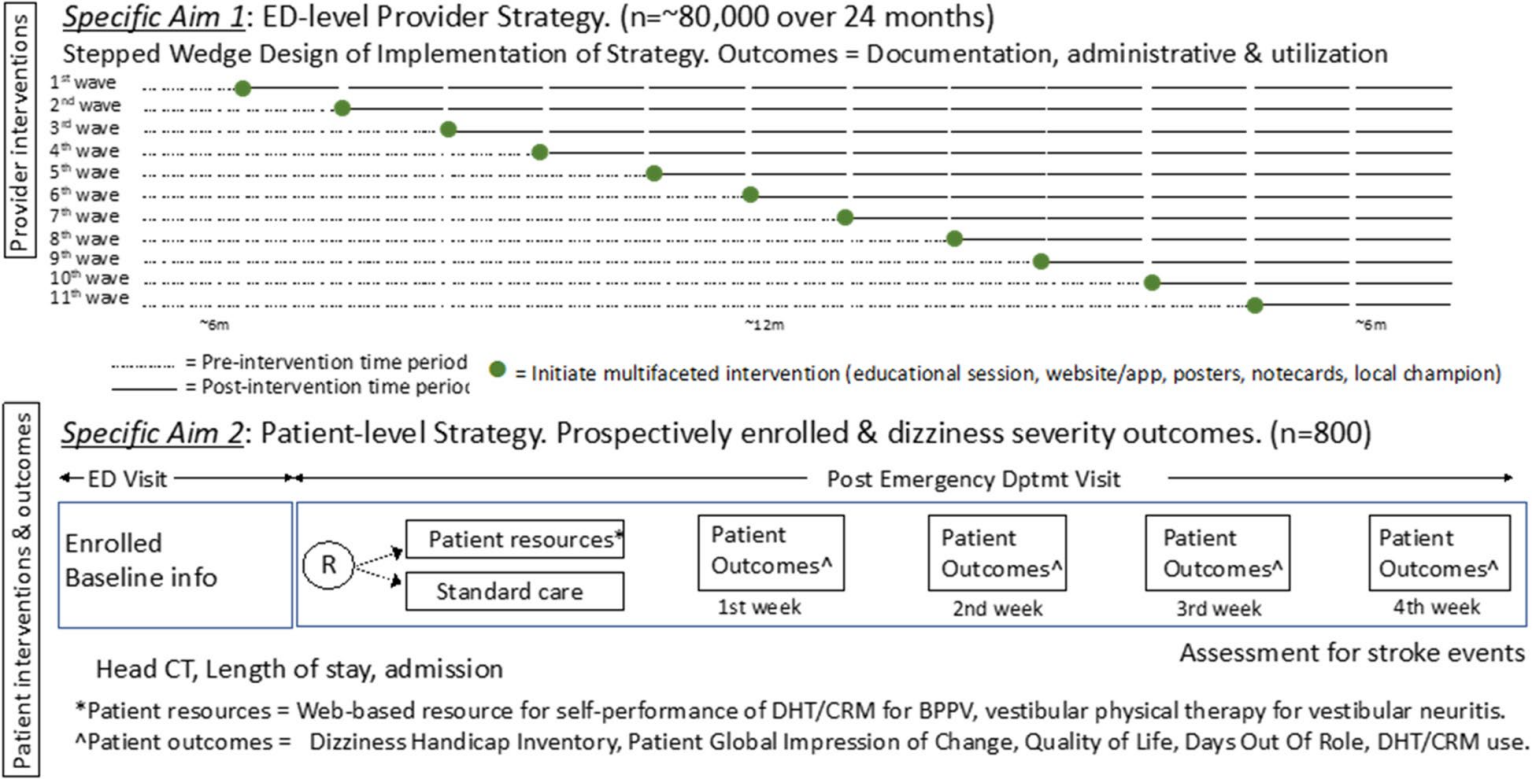
An overview of the timeline of ED (specific aim 1) and participant level (specific aim 2) interventions

Figure 2 depicts an overview of the timeline of ED (specific aim 1) and participant level (specific aim 2) interventions.

### Sample size {14}

#### ED level

The Kaiser Permanente Southern California study site is comprised of 14 ED facilities, which collectively had nearly 40,000 dizziness visits in 2017. We plan to collect data on these visits for approximately 24 months. Thus, for the ED-level strategy, we will evaluate documentation of the DHT and CRM in >80,000 dizziness visits anticipating an increase in volumes over time. We hypothesize that the proportion of target visits with DHT/CRM documentation will increase from 7% in the pre-intervention period to 17% in the post-intervention period based on the findings in our estimated target population in DIZZTINCT-1. Even small absolute increases in the frequency of use of the DHT/CRM in the target population are considered clinically meaningful because the DHT and CRM are simple from a resource standpoint, not expensive, low risk and highly effective in clinical trials. With a sample of 80,000 visits, our power to detect an increase in DHT/CRM use from 7% in the control visits vs 17% in the intervention visits will be effectively 100% based on a two-sided test for independent proportions considering clustering at 14 sites, an intraclass correlation coefficient (ICC) by provider of 22% for provider-level variability (from DIZZTINCT-1) and a significance level of 5% (Table 1). We will use a mixed effects model to account for secular trends, hospital- and provider-specific features, and the potential relative association of the various components with the outcome. We estimate that 20% of the annual sample is the target population (80,000 × 0.20 = 16,000 visits) based on best available evidence that showed about 20% of dizziness patients in a national sample have BPPV characteristics. Of the 16,000 visits, approximately 8000 will be pre-intervention visits and 8000 post-intervention visits. With these volumes, we expect 560 (8000 × 7%) visits with DHT/CRM documentation in the pre-intervention period and 1360 (8000 × 17%) in the post-intervention period. Thus, we anticipate at least 1900 visits with DHT/CRM documentation. For modeling purposes, we should be able to easily meet and exceed the general rule of having 10 outcomes for each independent variable to avoid over-fitting. The large number of visits and anticipated outcomes will allow us to more closely examine differences at hospitals and provider types, and the relative influence of the various intervention components. We will be able to monitor use of the CME session, website, app, and reminder system and incorporate these variables in the model specific to the provider. The additional power will allow us to examine differences at hospitals and provider types, and the relative influence of the various ED-level implementation strategies.

**Table 1:** Power calculations for ED-level stepped wedge design

Control, proportion outcome*	Intervention, proportion outcome*	Alpha	ICC**	Power for 80,000 visits	Minimum sample size for power >0.8
7%	17%	0.05	22%	0.999	468
7%	12%	0.05	22%	0.999	2028
7%	9.5%	0.05	22%	0.999	7800

#### Patient level

##### Initial design

We planned to have 800 patients for analysis of the patient level intervention. We have two concurrent interventions: provider-based intervention (pre-intervention period vs post-intervention period) and patient-based intervention (treatment materials vs standard of care materials). As a result, there will be 4 different groups each with 200 participants: no intervention, provider-based intervention only, patient-based intervention only, both provider- and patient-level interventions. Consistent with 2019 extension of CONSORT guidelines for multi-arm parallel-group trials, [25] our power considerations are based on our two primary comparisons: no intervention vs ED-level intervention,no intervention vs patient-level intervention. To assess power, we consider a clinically meaningful effect size to be a >=15% difference in the DHI outcome between the intervention and control group. In the medical literature, there has been some variation in thresholds of the minimal clinically important change (MIC) for the DHI. In the original derivation of the DHI, an 18-point difference was felt to be the MIC [23]. However, this conclusion was based on standard errors of the difference from test-retest in 14 participants. In a subsequent study of 32 dizziness patients, Tamber et al. found that a change of 11 points (corresponding to a 28% difference) was a strong discriminator of patients who improved by at least 2 points on a global self-report disability measure (the Disability Scale) at 48 h compared with patients who did not improve (c-statistic, 0.83) [51]. Friscia et al. performed a study in 45 dizziness patients and found that an approximately 10% change in the DHI score after 4–6 weeks was strongly associated with the established minimal clinically important difference in the 15-item Global Rating of Change (GROC) scale (c-statistic, 0.80) [14]. In an outpatient clinical trial of an internet-based intervention for vestibular physical therapy and cognitive behavioral coping strategies in patients with chronic dizziness, Geragherty et al. found that the intervention group had a 14% lower DHI compared with the control group [16]. Supporting that the 14% difference is clinically meaningful, 62% of the intervention group reported overall improvement compared with 33% of controls (*p*<0.001) [16]. For DIZZTINCT-2, we concluded that it was best to ensure that we have power to detect the lower range of a minimal clinically important difference between groups (i.e., 15%) for two main reasons: (1) Given that our interventions are low risk, scalable and applicable to a large target population, even a relatively small effect size would likely justify wide implementation and (2) If our intervention also improves the efficiency of care, then even a very small improvement in clinical outcomes would also justify wide implementation.

For our power calculation, we expect the baseline DHI in these acute ED dizziness presentations to be approximately 60 [2, 17, 18, 22, 24, 34, 37, 48, 52]. In the short-term follow-up period, we expect the non-intervention groups to improve by approximately 30% [6, 52], corresponding to a mean outcome DHI of 42. We consider the MIC for the intervention groups compared with the control group to be 15%, corresponding to a mean outcome DHI of 35.7. A more than 15% difference will exceed Jacobson’s original threshold of change in baseline of 18 points for the intervention group,70 and also differ from the control group by the ~10% threshold identified by Friscia et al as an MIC [14]. Based on the medical literature, we expect the standard deviation (SD) of DHI outcomes in our population will be 20–50% of the mean [18, 37, 52]. For our power calculation, we use a SD of 16 which is the high end of the estimate.

To calculate power for the patient-level intervention, we used two-sample means test and adjusted the estimate considering the effective sample size accounting for the ICC of the ED-facility that we found in DIZZTINCT-1 (1.9%). For this comparison, we have a power of 0.97 to detect a 15% difference in DHI outcome (35.7 vs 42) with our target sample size of 200 per group (Table 2). To achieve a power of 0.80, we would only need 113 patients per group. If larger differences in the intervention and control groups are obtained, then our power increases accordingly.

**Table 2:** Power calculations for patient-level intervention differences in dizziness handicap inventory outcome in intervention vs control population

Control, mean outcome (DHI*)	Intervention, mean outcome (DHI*)	Outcome difference b/t treated and control	SD	Alpha	ICC**	Power for 400 subjects (200 per group)	Minimum sample size for power >0.8
42	31.5	25% better	16	0.05	1.9%	1.0	96 (48 per group)
42	33.6	20% better	16	0.05	1.9%	0.99	136 (68 per group)
42	35.7	15% better	16	0.05	1.9%	0.97	226 (113 per group)
30	25.5	15% better	12	0.05	1.9%	0.95	246 (123 per group)
20	17	15% better	8	0.05	1.9%	0.95	246 (123 per group)

Shaded row indicates the power calculation for the prespecified threshold of a 15% difference between intervention and control groups. Calculations made using two sample means test accounting for ICC of 1.9%

*DHI dizziness handicap inventory

**ICC intraclass correlation coefficient

ICC for Aim 2 power patient-level intervention is at the ED-level since this is where more clustering will occur in Aim 2.

To calculate power for how the ED-level intervention impacts the patients enrolled in the trial (and thus contributing a DHI, in contrast to the larger cohort of patient only contributing data from the electronic health record) (Table 2), our calculations were made by including the number of clusters (12 ED facilities) and cluster heterogeneity using the ICC (1.9%) (Table 3). For this comparison, we have a power of 0.89 to detect a 15% difference in DHI outcome (35.7 vs 42) with our target sample size of 200 per group (Table 3). To achieve power of 0.80, we would only need 132 patients per group.

**Table 3:** Power calculations for ED-level intervention differences in dizziness handicap inventory outcome in intervention vs control population

Control, mean outcome (DHI*)	Intervention, mean outcome (DHI*)	**Outcome difference b/t treated and control**	**SD**	**Alpha**	**ICC**	**Power for 400 subjects (200 per group)**	**Minimum sample size for power >0.8**
42	31.5	25% better	16	0.05	1.9%	1.0	96 (48 per group)
42	33.6	20% better	16	0.05	1.9%	0.99	144 (72 per group)
42	35.7	15% better	16	0.05	1.9%	0.89	264 (132 per group)

Shaded row indicates the power calculation for the prespecified threshold of a 15% difference between intervention and control groups. Calculations made considering stepped wedge design at 15 sites accounting for ICC of 1.9%

*DHI dizziness handicap inventory. *ICC* intraclass correlation coefficient. ICC for Aim 2 power patient-level intervention is at the ED-level since this is where more clustering will occur in Aim 2

The additional power protects us against unexpected problems with recruiting or retention and also allows for additional exploratory analyses of differences in benefit based on symptom and patient characteristics.

##### Sample size revised

After initiating a process change allowing patients to self-enroll into the study, recruitment in the patient level intervention accelerated. The trial leadership, in consultation with the independent medical monitor and a representative from the sponsoring institute, elected to increase the maximum sample size for this group, to ensure adequate sampling before and after the provider level intervention in each site. The increased size also allows for increased power in important subgroups (fully specified in the statistical analysis plan). Patients with either BPPV or vestibular neuritis may represent as little as 20% of the overall cohort and we anticipate most of the benefit of the patient level intervention will be in these groups. Finally, we felt that there was a low risk of having excess power. The risk of demonstrating a numeric difference in the DHI that is not clinically relevant was considered low. In addition, the non-response patterns of participants across the 4 weeks of follow up surveys for the DHI introduce another issue that could reduce the effective power of the trial. This operational decision to increase sample size was made by the trial leadership, who did not have access to the outcome data by treatment assignment at the time of the decision. Based on enrollment projections, the maximum allowed sample size was set at 2000 (based on the plan for 6 months of recruitment following the last site level intervention), although the most likely final sample size is around 1500. In addition, the standard deviation of the DHI was higher than expected—therefore power was likely lower than our pre-trial assumptions.

### Recruitment {15}

Consistent with the goals of a large, scalable, pragmatic clinical trial, there are no research personnel on site to recruit patients. Instead, once a potentially eligible participant is identified, the patients will be contacted by study staff who will introduce the study to the patient, initiate the screening process for potentially eligible participants, and engage eligible and interested patients in the consent process. We will supplement our recruitment outreach efforts with text-based and email-based outreach. These additional outreach efforts are especially important recruitment adjuncts during the COVID-19 pandemic. This text-based approach will adhere to best practices in texting, such as allowing users to reply STOP if they do not want to be contacted again. We will have a bilingual staffed research team to answer any questions patients might have during the enrollment process.

## Assignment of interventions: allocation

### Sequence generation {16a}

#### ED level

The overall study period will be approximately 24 months. After the 6-month pre-intervention period, the sites will receive the intervention in 11 waves. The order will be determined by using a random number generator. The intervention start date at each ED is the date the physicians are auto enrolled for the online CME and the printed education materials (i.e., posters, notecards) are distributed on site.

#### Patient level

Participants will be randomized individually, stratified by site, sex and age, to either the usual care (control) or enhance usual care (intervention) arms using a random number generator. Twenty-five percent of enrolled participants will be assigned to each of the 4 arms. The allocations were generated in randomly permuted block sizes for each of the strata, to limit the possibility of study staff being able to predict the next assignment. Based on the time and hospital they present to for their dizziness care, the site may or may not have received the intervention.

### Concealment mechanism {16b}

We use the randomization module of REDCAP. Participants using self-enrollment and study staff enrolling patients over the phone are unable to view the assignment prior to randomization.

### Implementation {16c}

The sizes of the randomly permuted blocks were generated by the unblinded statistician and the study recruiting staff and principal investigators were unaware of the specific details.

## Assignment of interventions: blinding

### Who will be blinded {17a}

Sites are not blinded—but they do not receive the educational materials prior to the time point specified. The individual patient participants are unaware of whether the site has received the intervention. The individual patients will receive some information from a website (either the standard educational material on dizziness or the augmented self-treatment intervention website) so they are partially blinded. The clinical investigators are blinded to response on DHI (primary patient level intervention) by treatment group, however, do review adverse events with knowledge of treatment group assignment. The data analysts are not blinded.

The participants enrolled in the patient level intervention will have additional blinding to the assigned group because both groups will receive a link to a website of educational materials. The control group will receive a link to previously developed website resources about dizziness. The intervention group will receive a link to the study’s patient educational website.

### Procedure for unblinding if needed {17b}

Not applicable.

## Data collection and management

### Plans for assessment and collection of outcomes {18a}

Enrolled participants will complete the dizziness handicap inventory survey to obtain baseline dizziness disability. Participants will be asked to complete weekly surveys for 4 weeks to measure dizziness handicap over time. The close follow-up will protect against loss to follow up. Computerized surveys will be used at follow up to assess patient reported outcomes. Generally, the participants will complete on their own on a computer or smartphone. The participants baseline survey includes the following elements.

- Dizziness characteristics: Including questions about the type of dizziness, duration, severity, triggers, and associated symptoms.
- Dizziness handicap inventory: Identifies dizziness disability and includes functional, emotional, and physical elements.
- Days out of role: self-report measure used by the World Health Organization to quantify the number of days a person was totally unable to carry out usual activities because of a health condition
- Patient Health Questionnaire (PHQ)−4
- Patients will also be asked if a DHT or CRM was performed at or since the ED visit, when it was performed, and who performed it (self, provider, other)

### Plans to promote participant retention and complete follow-up {18b}

We will use various methods to ensure the retention of participants that have been shown to be effective for us in prior trials. At enrollment, the study team collects as much contact information as possible from the subject. We will also make obtaining the outcome information as easy and convenient as possible for subjects. Subjects will have several options for completing the outcome assessment including phone interview, text messaging response, or online survey in either English or Spanish We will send subjects notifications for the outcome assessment using phone calls, emails, and text messages. With careful attention to these additional retention methods, high rates of retention should be as good or better in this project as compared to our prior studies.

### Data management {19}

Research data for eligible patients will be captured electronically via electronic medical records. Additionally, we will obtain data directly from patient questionnaires. We will use a REDCap database to screen and conduct outreach to potentially eligible patients. The REDCap database will be housed within KPSC. The data and safety monitoring plan for this study included monitoring recruitment progress and potential adverse events resulting from data collection and the intervention activities. This research study is staffed by a research support team with a project manager and data manager/programmer to establish and monitor all data management protocols. All data will be abstracted into electronic data files that will use a subject code to identify participants. These subject codes will be linked to patient medical record number and name in a file separate from the data files. Data will be stored on password protected, encrypted environment with user-level access control limited to study personnel. All data transfers are encrypted. Access to any hard copy data will be restricted to internal research team members and stored in locked file cabinets.

### Confidentiality {27}

Breach of confidentiality and risk of psychological distress: We are mindful of the sensitive nature of patient’s medical records and have the utmost concern for the human subjects who will be part of this study and will take steps to ensure protection of confidentiality. Confidentiality of data will be maintained in this study through several standard procedures. Electronic data will be kept in password-protected files on a secure network at KPSC. Subjects will be identified through a unique identification number. This number will be used for all forms related to this study and in the analytic datasets. Thus, the identity of the person providing data cannot be determined from the raw data themselves. A master file containing the names of participants and their unique identifiers will be kept in a password-protected computer file, separate from the analytic files. All research study staff with access to protected health information have been trained in procedures to protect the confidentiality of subject data.

Analytic files will be maintained in a secure, password-protected environment with user-level access control limited to study personnel.

### Plans for collection, laboratory evaluation and storage of biological specimens for genetic or molecular analysis in this trial/future use {33}

We will not collect biological specimens as part of this trial.

## Statistical methods

### Statistical methods for primary and secondary outcomes {20a}

The primary outcome is the dizziness handicap inventory (DHI) which is a continuous (0–100) measure of dizziness disability based on a patient’s responses to 25 survey questions. Higher scores indicate more disability. We will use the Ceballo validated Spanish translation for the Spanish speaking individuals. As a sensitivity analysis, we will substitute 2 Lopez questions because we did not think the Ceballo translation was optimal for the following questions: Does bending over increase your problem? (Ceballo: When you get up, does your problem increase?]-Does turning over in bed increase your problem? (When you get out of bed, does your problem increase?] The DHI outcome will be obtained at 1-week intervals after the ED visit for 4 consecutive weeks—corresponding to 4 assessments. We will obtain 4 weekly assessments as we expect that time to recovery will vary given our heterogenous dizziness population. The primary analysis will be a multi-level model as follows: dependent variable (outcome) is the DHI (continuous); fixed independent variables are ED-level intervention group (0/1), patient intervention group (0/1), an interaction term of ED-level intervention with patient intervention group, patient severity at the time of ED visit (continuous), and assessment number (categorical, 1–4); and including a random ED-level intercept. This method allows us to capture variability in recovery over time while maintaining all of the variation in our continuous outcome. We include the severity at the time of the ED visit measure in the model to adjust for baseline disability, protecting against any random imbalances. Because of the two concurrent interventions (ED-level [0/1], patient-level [0/1]), patients will be in either a no intervention group, an ED-level intervention group only, a patient-level intervention group only, or both an ED- and patient-level intervention group. For a secondary analysis, we will add an interaction term of assessment number and intervention group (i.e., a 3-way interaction term of ED-level intervention, patient-level intervention, and assessment number) to further evaluate if any association of intervention group with the DHI outcome is modified by time to assessment. We will make comparisons of the effectiveness of the intervention at each time interval by estimating average marginal effects from our secondary model and comparing marginal effects using Wald Tests. We do not anticipate that our interventions will have differential effects by sex, either at the patient or provider level. Dizziness presentations in the emergency department are more common in females, although at this point there is little biological understanding of the reason for this. For the patient level analyses, we will disaggregate the data and perform analyses separate for male and female patients to assess for heterogeneity. In secondary analyses, we will explore if patient factors (age, sex, Charlson co-morbidities, health literacy) modify the effect of interventions with the outcome. Health literacy will be measured at the first outcome assessment using the BRIEF health literacy tool, 80 which was selected using the Boston University Health Literacy Tool Shed.

We initially considered performing the analysis using time to event (Cox proportional hazard model) based on a fixed and pre specified percentage improvement in the DHI from baseline. We felt, however, that this approach had two important limitations. First, there is not an established DHI threshold of success to use for time to event, and the appropriate threshold likely varies by condition. Second, a time to event analysis would dichotomize a continuous variable which substantially comprises statistical efficiency, information, and precision (e.g., true difference in degree of DHI improvement), and power [12, 36, 47, 49].

SPIRIT guidance: Statistical methods for analyzing primary and secondary outcomes. The statistical analysis for secondary and exploratory outcomes will be multi-level regression models as with our primary outcomes. For the cost analysis, the inputs will include tests, medications, procedures, consultations, and level of visit. Additional costs will also be gathered relating to hospital admission and subsequent visits to the ED. Unit costs will be derived from nationally representative sources (e.g., Medicare fee schedule). Costs often have a skewed distribution and thus will be log-transformed to achieve a more normal distribution. The costs in patients treated by pre-intervention providers will be compared to costs in patients treated by post-intervention providers using the t-test and also a generalized linear model (GLM). General concepts for modeling costs will be followed. [40, 46] Reference to where other details of the statistical analysis plan can be found, if not in the protocol.

### Interim analyses {21b}

No interim analyses are planned.

### Methods for additional analyses (e.g. subgroup analyses) {20b}

Full plans for subgroup analyses will be codified in the statistical analysis plan prior to database lock.

### Methods in analysis to handle protocol non-adherence and any statistical methods to handle missing data {20c}

Many participants will not complete all 4 surveys; however, the longitudinal models planned for the primary analyses above should account for this. When appropriate, multiple imputation will be used. Exploratory analyses may focus on how many physicians at a site used the CME intervention and if possible, we will conduct analyses evaluating the primary outcome within the subset of physicians who viewed the CME.

### Plans to give access to the full protocol, participant level-data and statistical code {31c}

The protocol and statistical code will be available online after publication of the main manuscript. Participant level data (with identifiers removed) may be available, under the conditions of sufficient funding by a qualified requester trained in human subject confidentiality protocols and with proper data use agreements between all institutions that govern data access, story and short- and long-term use.

## Oversight and monitoring

### Composition of the coordinating centre and trial steering committee {5d}

The coordinating center will be comprised of the multiple PIs and key co-investigators from neurology, stroke, physical therapy, health intervention design, and biostatistics. The multiple PIs in conjunction with the project manager will meet weekly with the enrolling and follow-up staff to coordinate the day-to-day operation of the trial.

### Composition of the data monitoring committee, its role and reporting structure {21a}

In accordance with sponsor guidelines and low risk level of study, a single independent medical monitor (IMM) with expertise in neuro-otology oversees the safety of the trial. The IMM regularly reviews the protocol, accrual information, outcomes and adverse events by treatment group and provides advice to the investigators and the sponsoring institute regarding trial continuation.

### Adverse event reporting and harms {22}

An adverse event (AE) is any unfavorable and unintended sign (including a clinically significant abnormal laboratory finding), symptom, or disease temporally associated with the use of a medical treatment or procedure regardless of whether it is considered related to the medical treatment or procedure (attribution of unrelated, unlikely, possible, probable, or definite). Each AE is a unique representation of a specific event used for medical documentation and scientific analysis.

A serious adverse event (SAE) is an AE that is fatal or life threatening, is permanently or substantially disabling, requires or prolongs hospitalization, results in a congenital anomaly, requires intervention to prevent permanent impairment or damage, or any event that the treating clinician or internal medical monitor judges to be a significant hazard, contraindication, side effect, or precaution. Reporting serious adverse events (SAEs) are based on the guidelines of the International Conference on Harmonization (ICH).

For the purposes of the trial, we will only capture patient level AEs and SAEs that are possibly, probably or definitely related to the performance of BPPV care processes (Dix-Hallpike test or Canalith Repositioning Maneuver).

Events will be captured for the ED focused intervention by screening the medical records using natural language processing complemented by manual review of visits with DHT/CRM documentation, and subsequent medical visits with the primary diagnosis of the adverse events. For the patient facing intervention, adverse events will be captured using the same mechanisms as above, plus additional capture from patient surveys at the outcome assessment. The principal investigators are responsible for designating, at the time an AE is reported, how likely it is that the AE was caused by the study intervention. These decisions (classification of relatedness) will be reviewed by the IMM. This determination requires clinical judgment, but for purposes of this study an algorithm is used to help the investigators provide reporting that is as objective as possible and consistent with reporting across the trial.

### Frequency and plans for auditing trial conduct {23}

The statistical team frequently reviews data from the trial and performs validity and outlier checks.

### Plans for communicating important protocol amendments to relevant parties (e.g. trial participants, ethical committees) {25}

The clinical coordinating center will propose any protocol amendments first to the institute and the IMM. After feedback and approval from these bodies, the IRB of record will receive a protocol amendment via their submission system.

### Dissemination plans {31a}

The primary dissemination plan is via publication of the main manuscript. Should the intervention be effective, KPSC plans to consider making elements of the physician and patient facing resources persistent and available to their members. The results will be posted to clinicaltrials.gov in accordance with the requirements of the National Institutes of Health.

## Discussion

The DIZZTINCT-2 trial addresses key areas of uncertainty in how to improve the care of emergency department patients with acute vertigo. The design incorporates a large, pragmatic, health system focused intervention along with a smaller, patient focused self-care intervention in a stepped-wedge design with an embedded individually patient randomized clinical trial. The first DIZZTINCT trial demonstrated that ED physicians could be intervened upon to do more physical examination (DHT and CRM) and less low value imaging. Major areas where more data was needed following the first DIZZTINCT trial included patient-oriented outcomes, along with tools to address diagnoses other than benign positional vertigo such as vestibular neuritis and migraine. In addition, follow up data on how much and how fast patients improved was needed. DIZZTINCT-2 will address these key knowledge gaps efficiently.

## Trial status

The trial is operating under Protocol Version 3. Patient enrollment started in November 2022—and the last participant included was enrolled in March 2025. The last follow up of secondary outcomes was anticipated in June 2025 (because not all stroke cases would be immediately coded—the full data for this secondary outcome measure was not available until later in summer 2025). The health system focused aim included patients before and after the individually randomized patient focused aim. This manuscript was delayed in submission due to attempts to train generative artificial intelligence (AI) large language models to reformat the scientific protocol document, based on prior Trials publications where there were also publicly available scientific protocols. The AI model made promises, and at times would work for several weeks noting it was close to producing an algorithm that could accomplish this. It turns out it was incorrect. The first author would recommend against using AI models to reformat scientific protocols into the Trials SPIRIT template.

## Data Availability

The multiple principal investigators will have access to the final trial dataset. Access of this dataset to others will be governed by the sponsoring health system.

## Abbreviations

AE: Adverse event
BPPV: Benign paroxysmal positional vertigo
CI: Confidence interval
CME: Continuing medical education
CRM: Canalith repositioning maneuver
CT: Computed tomography
DHI: Dizziness handicap inventory
DHT: Dix-Hallpike test
ED: Emergency department
EMR: Electronic medical record
GLM: Generalized linear model
ICC: Intraclass correlation coefficient
ICH: International conference on harmonization
IMM: Independent medical monitor
IRB: Institutional review board
KPSC: Kaiser Permanente Southern California
MIC: Minimal clinically important change
NIH: National Institutes of Health
PHQ: Patient Health Questionnaire
PRO: Patient reported outcome
RCT: Randomized controlled trial
SAE: Serious adverse event
SD: Standard deviation
SPIRIT: Standard Protocol Items: Recommendations for Interventional Trials
